# Procyanidin A2 Modulates IL-4-Induced CCL26 Production in Human Alveolar Epithelial Cells

**DOI:** 10.3390/ijms17111888

**Published:** 2016-11-12

**Authors:** Sara L. Coleman, Marlena C. Kruger, Gregory M. Sawyer, Roger D. Hurst

**Affiliations:** 1 Food and Wellness Group, The New Zealand Institute for Plant & Food Research Ltd., Palmerston North 4474, New Zealand; greg.sawyer@plantandfood.co.nz (G.M.S.); roger.hurst@plantandfood.co.nz (R.D.H.); 2 School of Food and Nutrition, Massey Institute of Food Science and Technology, Massey University, Palmerston North 4442, New Zealand; m.c.kruger@massey.ac.nz

**Keywords:** airway inflammation, eotaxin-3 (CCL26), IFNγ, IL-4, procyanidin A2

## Abstract

Allergic asthma is an inflammatory lung disease that is partly sustained by the chemokine eotaxin-3 (CCL26), which extends eosinophil migration into tissues long after allergen exposure. Modulation of CCL26 could represent a means to mitigate airway inflammation. Here we evaluated procyanidin A2 as a means of modulating CCL26 production and investigated interactions with the known inflammation modulator, Interferon γ (IFNγ). We used the human lung epithelial cell line A549 and optimized the conditions for inducing CCL26. Cells were exposed to a range of procyanidin A2 or IFNγ concentrations for varied lengths of time prior to an inflammatory insult of interleukin-4 (IL-4) for 24 h. An enzyme-linked immunosorbent assay was used to measure CCL26 production. Exposing cells to 5 μM procyanidin A2 (prior to IL-4) reduced CCL26 production by 35% compared with control. Greatest inhibition by procyanidin A2 was seen with a 2 h exposure prior to IL-4, whereas IFNγ inhibition was greatest at 24 h. Concomitant incubation of procyanidin A2 and IFNγ did not extend the inhibitory efficacy of procyanidin A2. These data provide evidence that procyanidin A2 can modulate IL-4-induced CCL26 production by A549 lung epithelial cells and that it does so in a manner that is different from IFNγ.

## 1. Introduction

Procyanidins are polyphenolic secondary plant metabolites that are recognized as biologically active in the context of human health. They are the product of polymerization of monomeric flavan-3-ol units by a currently undefined mechanism in plants [1]. The monomeric units can be linked together in various patterns, which can then dictate their ability to influence specific biological pathways. Polyphenolic compounds are thought to have evolved within plants to assist in coping with physiological stresses such as drought, excess UV exposure and/or pathogenic invasion [2]. Furthermore, there is accumulating evidence that plant polyphenols may have a role in managing stress in the human body [3,4]. Unfortunately, the average consumption of fruit and vegetables per person in the US is half of the USDA recommended daily intake of 1.5–2 cups and 2–3 cups, respectively [5]. Thus, concentrated extracts or isolated bioactive plant compounds could supplement the typical western diet for improved health and wellbeing.

Inflammatory diseases, such as allergic asthma, are less prevalent in populations that consume procyanidin-rich diets [6,7]. The lung epithelium is responsible for regulating pulmonary inflammation and secretes a range of cytokines, which coordinate physiological responses that restrict airflow. Eosinophil migration into the lung tissue is a defining feature of the inflammation that perpetuates allergic asthma [8]. The eosinophil chemokine eotaxin-3 (CCL26) is responsible for sustaining eosinophil migration in the lung tissue for at least 24 h after allergen exposure [9].

**Figure 1:**
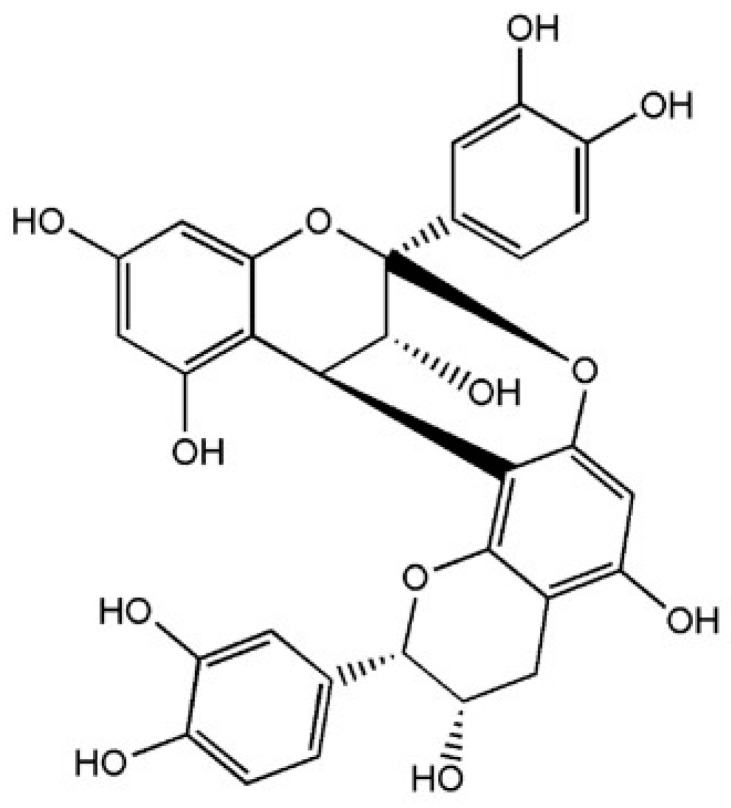
Procyanidin A2: epicatechin-(4β-8, 2β-*O*-7)-epicatechin.

Procyanidin A2 is one structurally defined A-type procyanidin (Figure 1) that is predominately found in cranberries and lingonberries. In this study, we investigated a commercially sourced, procyanidin A2 for in vitro efficacy in modulating interleukin-4 (IL-4)-induced CCL26 production relevant to allergic asthma. We characterized the concentration and temporal patterns of CCL26 inhibition by procyanidin A2. Interferon γ (IFNγ) is a cytokine with dichotomous roles as both an innate and adaptive immune modulator. It is pro-inflammatory in regard to autoimmune disease [10,11], and anti-inflammatory in regard to Th2 perpetuated disease, such as allergic asthma [12,13,14]. Investigation of IFNγ (100 ng/mL; 5.8 nM) in vitro has demonstrated ability to inhibit IL-4-induced CCL26 production from lung epithelial cells [12,15]; furthermore, IFNγ administered via the airways has demonstrated efficacy in mice at reducing airway eosinophilia [16]. Thus, we also evaluated the temporal profile of CCL26 inhibition by IFNγ as a tool for comparison and investigated possible interactions between IFNγ and procyanidin A2 seeking insights into how procyanidin A2 might modulate in vitro airway inflammation.

## 2. Results

### 2.1. Optimization of Airway Epithelial Cell Bioassay Conditions

**Figure 2:**
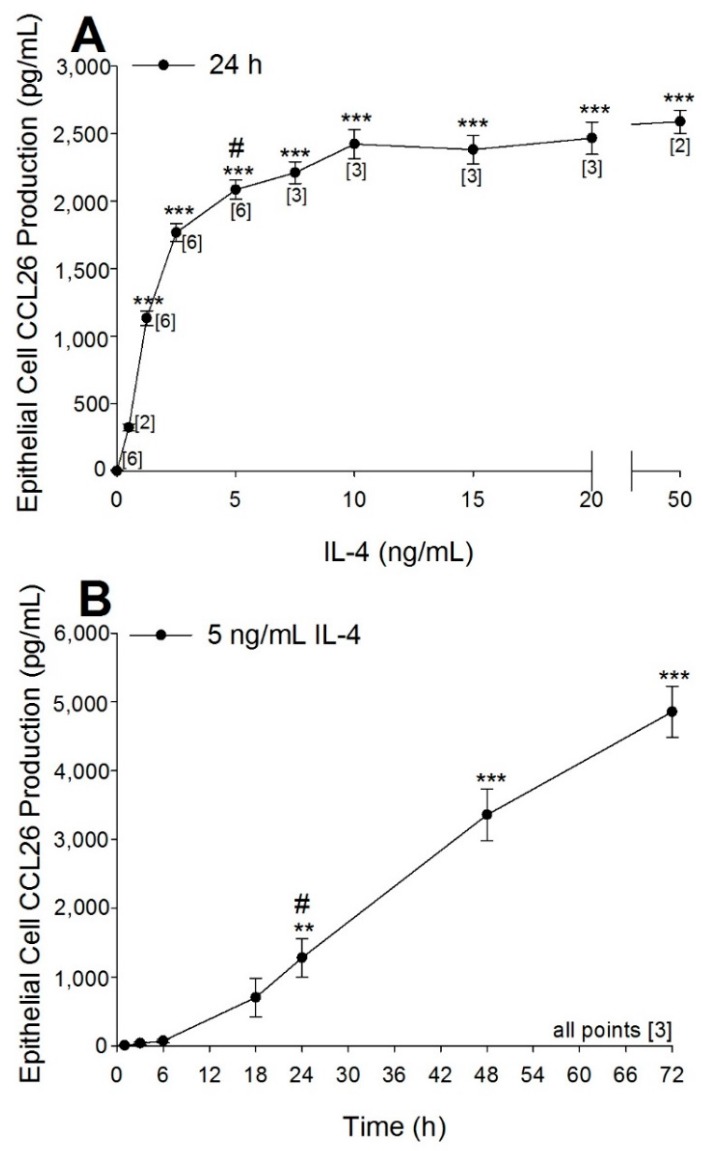
Optimization of IL-4 (interleukin-4) concentration and time for inducing CCL26 (eotaxin-3) production from A549 human lung alveolar epithelial cells. A549 cells were seeded at 5 × 10^5^ in 12-well plates, serum starved for 24 h and incubated with (**A**) 0.5–50 ng/mL IL-4 for 24 h or (**B**) 5 ng/mL IL-4 for 1–72 h. Collected supernatants were measured for CCL26 by ELISA. The results are expressed as mean ± SEM, which were from 2–6 separate experiments and 6–18 individual measurements. Brackets, [*n*], indicate number of experiments for each data point. # indicates the chosen optimized dose or time for inducing CCL26 production. ** *p* < 0.01, *** *p* < 0.001.

The concentration response characteristics for CCL26 production following IL-4 exposure (24 h) are shown in Figure 2A. Production of CCL26 increased steeply with increasing IL-4 concentration and reached a plateau at 10 ng/mL IL-4 (2423 ± 109 pg/mL CCL26). The CCL26 response was statistically different from control at IL-4 concentrations of 1.25 ng/mL (1134 ± 54 pg/mL CCL26, *p* < 0.001) and above. The time course for CCL26 production following 5 ng/mL IL-4 ranging from 1 to 72 h is shown in Figure 2B. Production of CCL26 became statistically different from baseline at 24 h (1280 ± 276 pg/mL CCL26, *p* < 0.01) and continued to increase in a linear manner until the final measured time point of 72 h (4865 ± 373 pg/mL CCL26, *p* < 0.001). From these data we determined that 5 ng/mL IL-4 for 24 h provided a sufficiently robust induction of CCL26, which enabled us to progress into utilising this model for the evaluation of procyanidin A2.

### 2.2. Cytotoxicity Assessment

**Figure 3:**
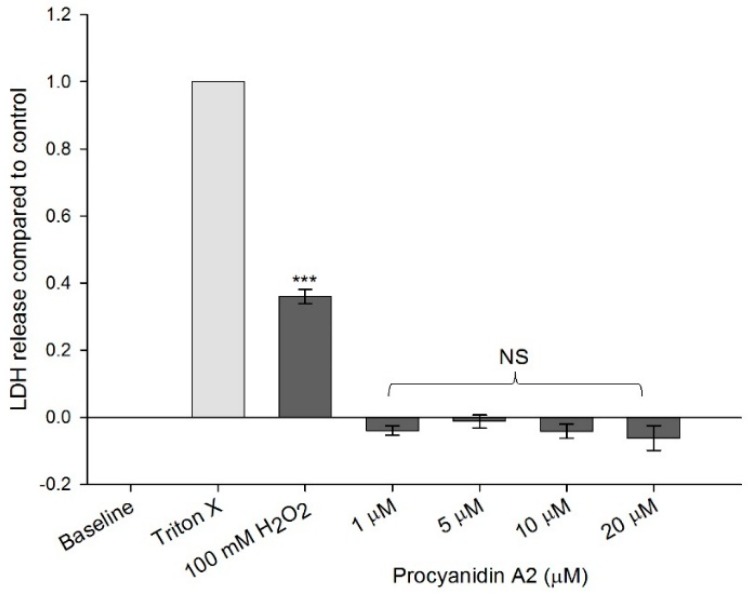
Effect of procyanidin A2 on cell viability, from lactate dehydrogenase assay. A549 cells were seeded at 9 × 10^5^ in 6-well plates, serum starved for 24 h and incubated with procyanidin A2 from 1 to 20 μM, control media, or positive control 100 mM H_2_O_2_ for 6 h. Supernatants were collected and assayed for lactate dehydrogenase (LDH). Results are expressed relative to baseline (0% cell death) and Triton X-100 (100% cell death). The results are expressed as mean ± SEM, which were from 3 separate experiments and 9 individual measurements. NS = not significant, *** *p* < 0.001.

To ensure any changes observed were due to procyanidin A2 and not an artefact of cytotoxicity, we evaluated the release of the cytosolic enzyme lactate dehydrogenase (LDH) from procyanidin A2-exposed epithelial cells as an indicator of the breakdown of the cellular plasma membrane. A positive control of 100 mM H_2_O_2_ was used, and demonstrated a measurable release of LDH (*p* < 0.001 compared with baseline control). Exposure to procyanidin A2 at 1, 5, 10 and 20 μM did not mediate any significant change in LDH release from the baseline control (Figure 3). Procyanidin A2 was therefore regarded as not cytotoxic to the epithelial cells at the concentrations and times evaluated.

### 2.3. Evaluation of Procyanidin A2

**Figure 4:**
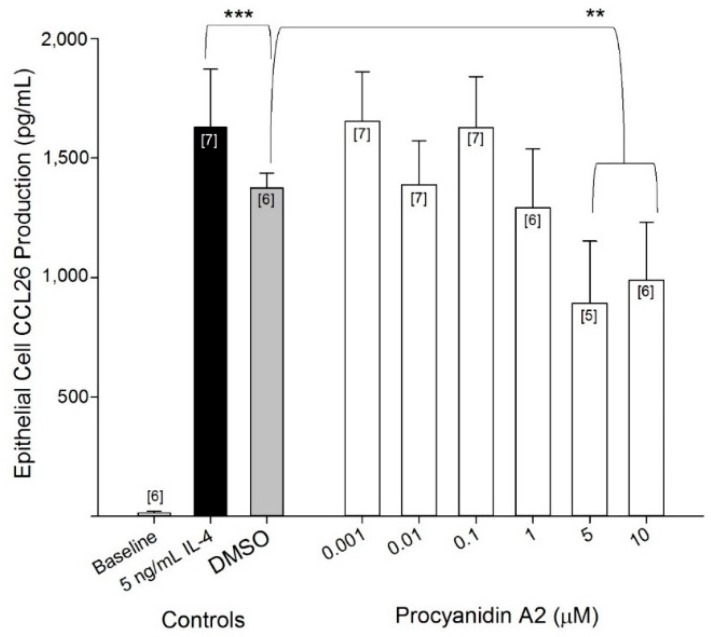
Procyanidin A2 inhibits IL-4-stimulated CCL26 production. A549 cells were seeded at 5 × 10^5^ in 12-well plates, serum starved for 24 h and incubated with control DMSO or a range of procyanidin A2 concentrations for 6 h, washed and then stimulated with 5 ng/mL IL-4 for 24 h. Collected supernatants were measured for CCL26 by ELISA. The results are expressed as mean ± SEM, which were from 5–7 separate experiments and 15–21 individual measurements. Brackets, [*n*], indicate number of experiments for each data point. ** *p* < 0.01, *** *p* < 0.001.

For experiments evaluating the potential modulation of CCL26 generation, we selected a procyanidin A2 concentration range of 0.001–10 μM. This procyanidin range was selected based on previous work from the literature, physiological relevance, and reported efficacy at inhibiting the secretion of another eotaxin isoform, CCL11 [17,18,19,20]. Procyanidin A2 at 5 and 10 μM were demonstrated to have significant inhibitory effects (891 ± 260 and 988 ± 242 pg/mL CCL26, respectively compared with the DMSO control 1375 ± 62 pg/mL CCL26; *p* < 0.01) on IL-4–induced CCL26 production when incubated for 6 h prior to an inflammatory insult of 5 ng/mL IL-4 for 24 h (Figure 4). These data corresponded to 35% and 28% inhibition of CCL26 production by 5 and 10 µM procyanidin A2, respectively beyond that of DMSO. Procyanidin A2 was dissolved in DMSO, which created different concentrations of DMSO in each investigated procyanidin A2 concentration. Control experiments were performed on the range of DMSO concentrations (to a maximum of 0.06%) corresponding to the anticipated doses of procyanidin A2. Only at the highest concentration evaluated was there a significant inhibitory effect of DMSO on CCL26 production from alveolar cells (1375 ± 62 pg/mL CCL26) compared with IL-4 stimulation only (1629 ± 242 pg/mL CCL26; *p* < 0.001; Figure 4). These data correspond to a 16% inhibition of CCL26 production by DMSO.

### 2.4. Time-Dependent Inhibition of CCL26 (Eotaxin-3) by Procyanidin A2 and IFNγ (Interferon γ)

**Figure 5:**
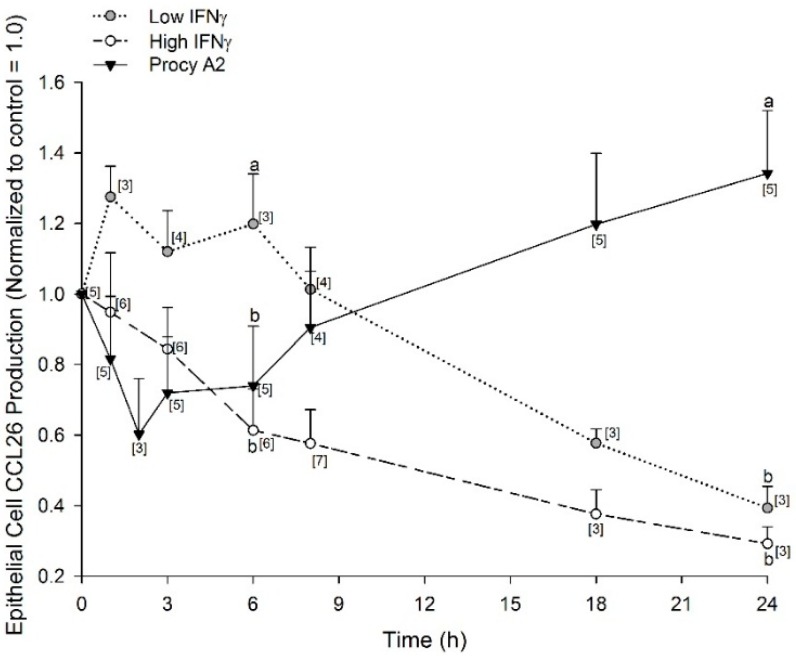
IFNγ and procyanidin A2 inhibit IL-4- stimulated CCL26 production in a time-dependent manner. A549 cells were seeded at 5 × 10^5^ in 12-well plates, serum starved for 24 h and incubated for 1–24 h with low IFNγ (0.5 nM, grey dots), high IFNγ (5.8 nM, open dots) or procyanidin A2 (Procy A2; 5 μM, black triangles), washed with PBS and then stimulated with 5 ng/mL IL-4 for 24 h. Collected supernatants were measured for CCL26 by ELISA. Results expressed as mean ± SEM normalized to control = 1.0, which were from 3–7 separate experiments and 9–21 individual measurements. Brackets, [*n*], indicate number of experiments for each data point. Different letters “a” and “b” at 6 h or 24 h represent statistical difference, *p* < 0.05.

The investigation of plant-based compounds as a means to reduce the reliance on pharmaceutical interventions and maintain human health is currently a very active research area. With evidence to support procyanidin A2 as a modulator of CCL26 production, we sought to explore the temporal pattern of CCL26 inhibition by procyanidin A2 and compared this with the known CCL26 secretion inhibitor, IFNγ. A time course of inhibition was conducted for 5 μM procyanidin A2 and compared against two concentrations of IFNγ: 0.5 nM (low) and 5.8 nM (high). The time courses for procyanidin A2, low IFNγ, and high IFNγ inhibition of CCL26 are shown in Figure 5. Procyanidin A2 data are expressed relative to control wells incubated with 0.03% DMSO for 24 h, or media only for IFNγ. Procyanidin A2 demonstrated peak inhibition of CCL26 production when incubated for 2 h (0.60 ± 0.16 normalized to control = 1.0) prior to an inflammatory insult. The inhibition was no longer observed at 8 h (0.90 ± 0.16; 24 h: 1.3 ± 0.18 normalized to control = 1.0; Figure 5). Low and high IFNγ demonstrated the greatest inhibition of CCL26 production when incubated for 24 h prior to an inflammatory insult with both concentrations significantly inhibiting to a similar degree (0.5 nM: 0.39 ± 0.06, 5.8 nM: 0.29 ± 0.05 normalized to control = 1.0; Figure 5). CCL26 production measured after 6 h exposure to the higher concentration of IFNγ was significantly different from that mediated in the presence of the lower concentration of IFNγ (0.5 nM: 1.20 ± 0.14, 5.8 nM: 0.61 ± 0.12 normalized to control = 1.0; *p* < 0.05; Figure 5). The low concentration of IFNγ did not inhibit CCL26 production to below control levels until after 8 h of exposure prior to the inflammatory insult. Visual inspection of A549 cells after incubation with 5.8 nM IFNγ (100 ng/mL) showed no disruption of the cells; the observed IFNγ-induced inhibition to 30% of control was consistent with reports in the literature [12,15].

A revealing feature of the CCL26 inhibition time courses is seen from the comparison of 5 μM procyanidin A2 with IFNγ concentrations at 6 h prior to an inflammatory insult. Procyanidin A2 inhibited CCL26 production to 74% of the control after 6 h, while low concentration IFNγ inhibited to 120% of the control, and high concentration IFNγ demonstrated an inhibition to 61% of the control. Thus 5 μM procyanidin A2 was a more potent inhibitor of CCL26 production at 6 h compared with low concentration IFNγ (procyanidin A2: 0.74 ± 0.16, 0.5 nM IFNγ: 1.20 ± 0.14 normalized to control = 1.0, *p* < 0.05). Procyanidin A2 had a similar inhibition of CCL26 production at 6 h when compared with high concentration IFNγ (5.8 nM IFNγ: 0.61 ± 0.12 normalized to control = 1.0; Figure 5). The concentration and time-dependent differences observed with procyanidin A2 and IFNγ concentrations lead us to consider possible cooperative interactions between procyanidin A2 and IFNγ.

### 2.5. Procyanidin A2 Impedes IFNγ-Mediated CCL26 Inhibition

**Figure 6:**
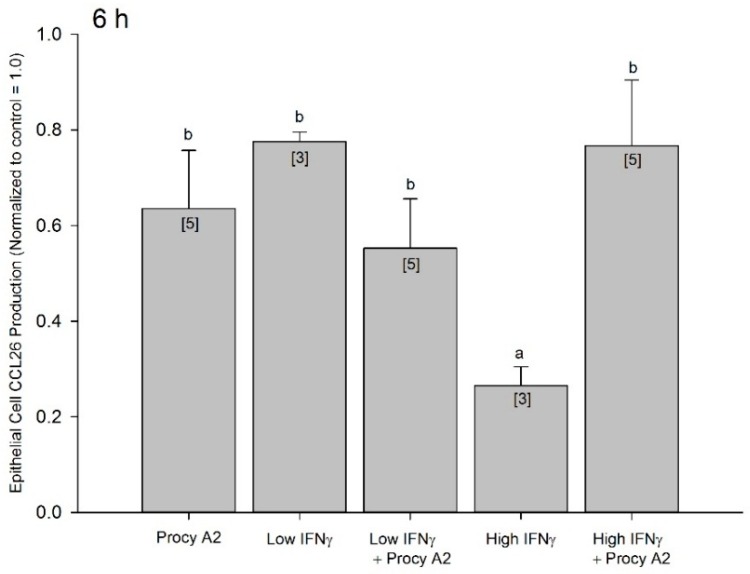
Concomitant incubation (6 h) of procyanidin A2 and IFNγ does not improve inhibition of CCL26 production. A549 cells were seeded at 5 × 10^5^ in 12-well plates, serum starved for 24 h and incubated for 6 h with either 5 μM procyanidin A2 (Procy A2), low (0.5 nM), high (5.8 nM) IFNγ alone, or concomitantly IFNγ plus 5 μM procyanidin A2. Cells were washed and then stimulated with 5 ng/mL IL-4 for 24 h. Collected supernatants were measured for CCL26 by ELISA. Results are expressed as mean ± SEM normalized to control = 1.0, which were from 3–5 separate experiments and 9–15 individual measurements. Brackets, [*n*], indicate number of experiments for each data point. Different letters “a” and “b” represent statistical difference, *p* < 0.05.

The distinct temporal patterns of CCL26 inhibition by procyanidin A2 and IFNγ prompted us to next evaluate the possibility that concomitant 5 μM procyanidin A2 and IFNγ may assist in maintaining peak procyanidin A2 inhibition (observed at 2 h) to a later (6 h) time point or improve inhibitory efficacy beyond that of procyanidin A2 alone (Figure 6). Investigating cooperative inhibition with a 6 h exposure prior to an inflammatory insult was chosen as both procyanidin A2 and IFNγ display suboptimal inhibition with this length of time. At 6 h the inhibition of CCL26 by 5 μM procyanidin A2 alone, low (0.5 nM) IFNγ, or the concomitant incubation of 5 μM procyanidin A2 with low IFNγ showed no statistical difference from each other (0.64 ± 0.12, 0.78 ± 0.02, 0.55 ± 0.10 respectively normalized to control = 1.0; Figure 6). High IFNγ alone significantly inhibited CCL26 production beyond that of procyanidin A2 (0.27 ± 0.04 normalized to control = 1.0; *p* < 0.05; Figure 6). When 5 μM procyanidin A2 was concomitantly added with high (5.8 nM) IFNγ, CCL26 production was no longer statistically different from procyanidin A2 alone (0.77 ± 0.14 normalized to control = 1.0; Figure 6). This data suggests procyanidin A2 does not have cooperative inhibition with IFNγ, and in fact interferes with high dose IFNγ-mediated inhibition of CCL26.

## 3. Discussion

This study sought to evaluate the potential modulation of cytokine-induced CCL26 by a commercially sourced, structurally-defined, pure dimeric procyanidin, procyanidin A2. We confirmed IL-4 exposure induces CCL26 production in A549 cells, thereby modelling an aspect of airway inflammation of relevance to allergic asthma. Exposure time and IL-4 concentration were optimized for CCL26 production. These experiments demonstrated that 5 ng/mL IL-4 for 24 h allowed for a robust induction of CCL26 production in A549 cells. Using these parameters we demonstrated that both 5 and 10 μM procyanidin A2 when incubated 6 h prior to an inflammatory insult had an inhibitory effect on CCL26 production. Experiments determining cytotoxicity by measuring LDH, along with previous work in our group using the WST-1 assay [18], demonstrate that procyanidin A2 at the concentrations investigated in this study were not detrimental to cell membrane integrity or cell metabolism; thus, the inhibitory effect observed on the inflammatory biomarker CCL26 was attributed to an action of procyanidin A2 and not cytotoxicity. We then expanded our investigation of procyanidin A2 by measuring the temporal profile of inhibition for 5 μM procyanidin A2 and used the known CCL26 secretion inhibitor IFNγ as a tool for comparison as well as investigated possible cooperative inhibition of CCL26 secretion by procyanidin A2 and IFNγ.

There is a growing pool of evidence that supports the ability of polyphenolic compounds, specifically procyanidins, to modulate key inflammatory events (see reviews) [3,4]. Aberrant inflammatory responses at the airway epithelium of an asthmatic prolongs immune cell presence in the lung leading to tissue damage. The eosinophil chemokine CCL26, which facilitates the late-stage infiltration of eosinophils into lung tissue, is one target for procyanidin modulation that could be useful for improving lung health [21]. We contribute evidence with our work here that plant polyphenol procyanidin A2 can indeed act as a modulator of inflammation. We provide evidence that procyanidin A2 inhibits IL-4-induced production of CCL26 in vitro and its efficacy is the greatest when exposed to cell cultures 2–3 h prior to an inflammatory insult. This temporal profile is distinct from that of the CCL26 inhibitor IFNγ, which demonstrated greatest inhibition in our model system at 24 h. IFNγ is a known effector of CCL26 production and can act on the epithelium to inhibit airway inflammation [16]. There are several mechanisms by which IFNγ has been demonstrated to inhibit CCL26 production, including through intracellular effects on transcription factors [22], up-regulation of endogenous inhibitors [23], increasing the rate of decay for CCL26 messenger RNA [12], increasing the degree of methylation in the promoter region of the *CCL26* gene [24], and increasing the expression of the IL-4 receptor component, IL-4Rα [15]. The temporal pattern of inhibition for procyanidin A2 reported here does not match that of IFNγ. The temporal profile of 5 μM procyanidin A2 inhibition was strongest at 2 h, reduced by 8 h, and was no longer present by 24 h (Figure 5). When we investigated possible coordinated interactions between procyanidin A2 and IFNγ, we found no evidence of cooperative inhibition. In contrast, procyanidin A2 interfered with high-concentration IFNγ-mediated inhibition of CCL26 when simultaneously incubated together prior to an inflammatory insult. These data allude to a more transient mechanism of inhibition by procyanidin A2 and likely via a mechanism of action independent from IFNγ.

To date the demonstrated biological effects of A-type linked procyanidins include activity as an antioxidant, which is attributed to the catechol group in the B-ring of many flavonoids that can trap free radicals [25,26,27], and in the prevention of oral and urogenital epithelial infections due to the anti-adherence properties [28,29,30]. The inclination of procyanidins to bind proteins and form insoluble complexes [31] leads to the possibility of direct cell receptor masking in our experiments; though we did wash cells prior to cytokine exposure as an attempt to prevent masking of the IL-4 receptor by procyanidin A2. The experiments presented here were performed with the intention of demonstrating procyanidin efficacy for modulating IL-4-induced CCL26, with less emphasis on determining the underlying mechanism. However, major signal transduction pathways such as signal transducers and activators of transcription (STAT), transcription factors in the mitogen-activated protein kinases (MAPK), as well as the NF-κB pathway have all been identified as being affected by the presence of procyanidins [4]; furthermore, controlling signal transduction pathways may not be the only mechanism by which procyanidins could influence inflammatory events. A more recent, and still developing, theory is that procyanidins could mediate a physical interaction at the cell membrane that modulates cellular events [32]. There is work in the literature that demonstrate other flavonoids and the flavan-3-ol monomeric units catechins, which bind to form procyanidins, can insert themselves in plasma membranes as a mechanism for eliciting biological activity [33,34,35]. Interestingly, experiments with liposomes suggested that procyanidins can protect against lipid oxidation by affecting membrane surface potential and integrity [25,36]. Extensive work by Verstraeten, Fraga, & Oteiza investigating A-type procyanidin effects on the gastrointestinal epithelium have demonstrated that they indeed can impact biological processes via interactions with the apical membrane of the enterocyte in vitro [32]. Procyanidins are indicated as mitigating oxidative stress and the activation of pro-inflammatory events [32]. Thus, it is suggested that A-type procyanidins may favourably affect gastrointestinal health, though there has been no work to date investigating A-type procyanidins and gut inflammation in vivo. Similarly, recent experiments by Zhu et al. have demonstrated membrane perturbations as a possible mechanism for the ability of A-type procyanidins to inhibit 3T3-L1 preadipocyte differentiation [37,38]. Our findings here, whereby procyanidin A2 interferes with an intracellular inhibitor IFNγ, is preliminary evidence that A-type procyanidins could interact with cellular membranes as a mechanism to reduce inappropriate inflammation and promote lung health.

Previous work from our group in a similar cell culture model suggested proanthocyanidins as the bioactive components responsible for the ability of blackcurrant extracts to modulate CCL26 production in vitro [39]. The study provided evidence to support the theory that a proanthocyanidin-enriched blackcurrant extract potentiated IFNγ-induced suppression of IL-4 stimulated CCL26 secretion, and the authors put forth the idea that proanthocyanidin metabolites, particularly epigallocatechin, may modulate similar cellular events and complement the inhibitory action of IFNγ on eosinophilic inflammation [39]. At first consideration, this appears to be in conflict with the data presented here; however the blackcurrant study used 1 ng/mL IFNγ (0.058 nM), which is consistent with our data for low (0.5 nM) IFNγ data presented here in which we show that procyanidin A2 trends (but did not reach significance) toward assisting low concentration IFNγ at inhibiting CCL26 production (Figure 6). The two studies differed in the duration of inhibition after exposing cells to polyphenolic compounds. The blackcurrant proanthocyanidin-enriched extract evaluated in the previous study displayed inhibition of CCL26 starting at 6 h, which remained evident at 24 h, whereas our work here demonstrated the strongest inhibition around 2 h and did not remain evident beyond 8 h. It may also be important to note that the blackcurrant work incubated cells with the proanthocyanidin-enriched extract prior to an inflammatory insult of IFNγ and IL-4 together, and our work here exposed cell to procyanidin A2 and IFNγ prior to an inflammatory insult of IL-4 alone. Furthermore, previous work in our group identified that the ratio of specific anthocyanins, another class of phytochemicals, in blackcurrant cultivars was an important determinant for influencing the suppression of cytokine-induced CCL26 in A549 cells [40]. Investigating the biological activity of polyphenols, and specifically procyanidins, has repeatedly indicated that structural specificity is paramount to bioactivity and it cannot be extrapolated across a phytochemical classification. For example, A-type linked and B-type linked procyanidins by virtue of structural differences demonstrate distinct interactions with the transcription factor nuclear factor-κB (NF-κB) [20]. Differences in bioactivity were also reported by our group between A-type and B-type procyanidins for inhibition of another isoform of eotaxin, CCL11 [18]. Furthermore, B-type procyanidins have been investigated by us for their ability to modulate IL-4-induced CCL26. Procyanidin B1 had no inhibitory effect, whereas procyanidin B2 had marginal inhibition compared to the effects shown by procyanidin A2 (Figure S1). Thus, the stronger inhibitory activity demonstrated by procyanidin A2 in the context here could be structure specific and/or restricted to polyphenolic compounds with A-type bonds between the flavan-3-ol monomeric units.

In order to progress our understanding of the role procyanidins may have in mitigating airway inflammation, future work could investigate procyanidin A2 by utilizing an air-liquid interface epithelial cell culture system. This would mimic and evaluate an application via inhalation as opposed to oral ingestion. Studies investigating the efficacy of IFNγ applied through inhalation have suggested its use in eosinophilic asthma, with effects limited to the respiratory tract [13]. This approach could also be applied to procyanidins. Furthermore, while we recognize more research is needed, our work here could suggest the potential for procyanidin A2 as a preventative measure for lung inflammation when exposure to known allergens is anticipated such as seasonal hay fever allergies, or air pollution, in addition to supporting therapeutic use for established disease.

## 4. Materials and Methods

### 4.1. Materials

The human alveolar epithelial (A549) cell-line was purchased from the American Type Culture Collection (ATCC^®^ CCL-185™, (c/o Cryosite, New South Wales, Australia)). Cell culture media, phosphate buffered saline (PBS), penicillin-streptomycin-neomycin antibiotic mixture, 100× l-glutamine, and 2.5% trypsin were purchased from Life Technologies (Auckland, New Zealand). Fetal bovine serum (FBS) was purchased from Moregate Biotech (Hamilton, New Zealand). Procyanidin A2 (HPLC ≥ 99%, epicatechin-(4β-8, 2β-*O*-7)-epicatechin) was purchased from Extrasynthese (Genay Cedex, France). The CCL26 DuoSet ELISA kit, human recombinant IL-4 and IFNγ were purchased from R&D Systems (Pharmaco, Auckland, New Zealand). Low-endotoxin bovine serum albumin was purchased from MP Biomedicals (Auckland, New Zealand). Ecoteric T20 (Tween 20) and 30% hydrogen peroxide were purchased from ThermoFisher Scientific (Auckland, New Zealand). Sodium acetate tri-hydrate was from BDH Laboratory Supplies (Poole, UK). β-nicotinamide adenine dinucleotide reduced dipotassium salt (NADH), dimethyl sulfoxide (DMSO) and all other chemicals not specifically listed were purchased from Sigma-Aldrich (St. Louis, MO, USA).

### 4.2. Cell Culture Conditions

Cells were grown under standard tissue culture conditions as previously described [18]. Briefly, A549 cells were keep at 37 °C in a 95% humidified atmosphere at 5% CO_2_ in Dulbecco’s Modified Eagle Medium: Nutrient Mixture F-12 (DMEM/F-12) containing 2 mM l-glutamine, 50 μg/mL penicillin, 50 μg/mL streptomycin, 100 μg/mL neomycin, and 10% FBS. The cell cultures were grown to form a monolayer (80% confluence) and then growth arrested for 24 h in the absence of FBS before conducting experiments. Experiments used A549 cells of passage 89–107.

### 4.3. Optimizing the Production of CCL26

The production of CCL26 can be induced by IL-4 in A549 airway epithelial cells in vitro to model the inappropriate inflammation that occurs during allergic airway inflammation. In order to optimize a bioassay for CCL26 production, we characterized both the concentration- and time-dependent profiles of CCL26 production after incubation with IL-4. We measured CCL26 production in A549 cells exposed to IL-4 at a range of concentrations from 0.25 to 50 ng/mL for 24 h, as well as determined the time course of CCL26 production after exposure to 5 ng/mL IL-4 for times ranging from 1 to 72 h. Experiments used A549 cells seeded at 5 × 10^5^ per well in 12-well plates with culturing and growth arrest as described above. Cells were then exposed to serum-free media containing IL-4. Extracellular media were collected and frozen at −80 °C until analysis for CCL26 using a commercially available sandwich ELISA kit following the manufacturer’s instructions.

### 4.4. Procyanidin Preparation

Procyanidin A2 was dissolved in 100% DMSO to 10 mg/mL and stored in aliquots at −80 °C until experimental use. Procyanidin A2 was then brought to the desired concentration in serum-free media and added immediately to cell cultures. Final DMSO concentrations were 0.03% and 0.06% (*v*/*v*) DMSO, respectively, for cultures exposed to 5 μM (2.88 μg/mL) and 10 μM (5.765 μg/mL) procyanidin A2. These represented the negative controls.

### 4.5. Cytotoxicity

The potential cytotoxicity of procyanidin A2 was evaluated by determining effects on cellular plasma membrane integrity through the measurement of the release of the cytosolic enzyme LDH. Experiments used A549 cells seeded at 9 × 10^5^ in 6-well plates in a 3 mL volume with culture and growth arrest as described above. Cells were incubated with procyanidin A2 at concentrations from 1 to 20 μM, negative control media, or a positive control of 100 mM H_2_O_2_ for 6 h. A representation of 100% release of cellular LDH was obtained by exposing untreated cells to 1% Triton X-100 for 10 min at room temperature. Collected supernatant samples were centrifuged at 12,000× *g* for 15 min and immediately assayed for the presence of LDH. In a 96-well plate, 100 µL of each sample was pipetted into a well, and using automated microplate injectors of a BMG Polarstar Omega Plate Reader (Alphatech), LDH substrate consisting of 3.8 mM pyruvate and 0.8 mM NADH was added to one well at a time and absorbance of NAD+ read at 340 nm for 2 min.

### 4.6. Modulation of CCL26

In experiments examining effects of procyanidin A2 on inflammation, A549 cells were seeded at 5 × 10^5^ per well in 12-well plates with culture and growth arrest as described above. Cells were then incubated with media containing procyanidin A2 at final concentrations ranging from 0.0001 to 10 μM, or corresponding DMSO concentrations (max 0.06%) for 6 h, washed briefly with PBS, fresh media replaced and then stimulated with 5 ng/mL IL-4 for 24 h to induce CCL26 production. Extracellular media were collected and frozen at −80 °C until analysis for CCL26 production by ELISA. A similar protocol was used for experiments using low (0.5 nM) and high (5.8 nM) IFNγ. A time course of CCL26 inhibition was determined for 5 μM procyanidin A2, low concentration (0.5 nM) and high concentration (5.8 nM) IFNγ, by exposing A549 cells for 1–24 h prior to the inflammatory insult of 5 ng/mL IL-4. CCL26 production was also investigated following a 6 h concomitant exposure to 5 μM procyanidin A2 added to low (0.5 nM) and high (5.8 nM) IFNγ. Procyanidin A2 and IFNγ concentrations were prepared separately and added together in the cell culture well.

### 4.7. Statistics

Statistical analysis of data was performed using GraphPad Prism 5 (San Diego, CA, USA) or GenStat: 14th Edition (VSN International, London, UK). Data in the IL-4 concentration response (Figure 2A) and time course (Figure 2B) were analyzed with a one-way ANOVA and post-hoc Dunnett’s multiple comparison tests against control 0 ng/mL IL-4 or time 0 h, respectively. Cytotoxicity data (Figure 3) were analyzed with a one-way ANOVA and post-hoc Tukey’s multiple comparison tests between all experimental conditions. Data for CCL26 inhibition by procyanidin A2 (Figure 4) as well as data for concomitant incubation of procyanidin A2 and IFNγ (Figure 6) were analyzed with a mixed linear model and post-hoc Fisher’s unprotected least significant difference test (5%) because the data were unbalanced. Time points of interest for data in Figure 5 were analyzed with one-way unpaired *t*-tests with a Welch’s correction. Data that did not reach a *p* < 0.05 threshold were listed as not significant (NS). Statistical significance in the figures is denoted as * *p* < 0.05, ** *p* < 0.01, *** *p* < 0.001.

## 5. Conclusions

Our data provides evidence that pre-exposure to procyanidin A2 is a prompt yet transitory method to inhibit CCL26 production in A549 human lung epithelial cells. The temporal profile of inhibition is observed to be different from that of IFNγ. These data are encouraging, but further investigation is needed in an animal model or whole body system to determine if procyanidin A2 could be utilized as a preventative approach to help manage airway inflammation.

## Supplementary Materials

Supplementary materials can be found at www.mdpi.com/1422-0067/17/11/1888/s1.

## Abbreviations

CCL26	eotaxin-3
DMEM/F-12	Dulbecco’s Modified Eagle Medium: Nutrient Mixture F-12
FBS	fetal bovine serum
IFNγ	interferon γ
LDH	lactate dehydrogenase
MAPK	mitogen-activated protein kinases
NADH	β-nicotinamide adenine dinucleotide reduced dipotassium salt
NK-κB	nuclear factor κ-light-chain-enhancer of activated B cells
STAT	signal transducers and activators of transcription
USDA	United States Department of Agriculture
WST-1	water soluble tetrazolium-1

## References

[1] Xie D.Y., Dixon R.A. (2005). Proanthocyanidin biosynthesis—Still more questions than answers?. Phytochemistry.

[2] Joven J., Micol V., Segura-Carretero A., Alonso-Villaverde C., Menendez J.A., Bioactive Food Components Platform (2014). Polyphenols and the modulation of gene expression pathways: Can we eat our way out of the danger of chronic disease?. Crit. Rev. Food Sci. Nutr..

[3] Martinez-Micaelo N., Gonzalez-Abuin N., Ardevol A., Pinent M., Blay M.T. (2012). Procyanidins and inflammation: Molecular targets and health implications. BioFactors.

[4] Coleman S.L., Hurst R.D., Sawyer G.M., Kruger M.C., Sullivan I. (2015). Fruit Procyanidins: Modulating Inflammation to Promote Health. Proanthocyanidins: Food Sources, Antioxidant Properties, and Health Benefits.

[5] Usual Dietary Intakes: Food Intakes, U.S. Population, 2007-10. http://epi.grants.cancer.gov/diet/usualintakes/pop/2007-10/.

[6] Singh A., Holvoet S., Mercenier A. (2011). Dietary polyphenols in the prevention and treatment of allergic diseases. Clin. Exp. Allergy.

[7] Nyanhanda T., Gould E.M., Hurst R.D. (2014). Plant-derived foods for the attenuation of allergic airway inflammation. Curr. Pharm. Des..

[8] Ishmael F.T. (2011). The inflammatory response in the pathogenesis of asthma. J. Am. Osteopath. Assoc..

[9] Hallstrand T.S., Hackett T.L., Altemeier W.A., Matute-Bello G., Hansbro P.M., Knight D.A. (2014). Airway epithelial regulation of pulmonary immune homeostasis and inflammation. Clin. Immunol..

[10] Skurkovich S., Skurkovich B. (2005). Anticytokine therapy, especially anti-interferon-γ, as a pathogenetic treatment in Th-1 autoimmune diseases. Ann. N. Y. Acad. Sci..

[11] Miller C.H., Maher S.G., Young H.A. (2009). Clinical use of interferon-γ. Ann. N. Y. Acad. Sci..

[12] Heller N.M., Matsukura S., Georas S.N., Boothby M.R., Rothman P.B., Stellato C., Schleimer R.P. (2004). Interferon-γ inhibits STAT6 signal transduction and gene expression in human airway epithelial cells. Am. J. Respir. Cell Mol. Biol..

[13] Martin R.J., Boguniewicz M., Henson J.E., Celniker A.C., Williams M., Giorno R.C., Leung D.Y.M. (1993). The effects of inhaled interferon-γ in normal human airways. Am. Rev. Respir. Dis..

[14] Flaishon L., Topilski I., Shoseyov D., Hershkoviz R., Fireman E., Levo Y., Marmor S., Shachar I. (2002). Cutting edge: Anti-inflammatory properties of low levels of IFN-γ. J. Immunol..

[15] Yamamoto S., Kobayashi I., Tsuji K., Nishi N., Muro E., Miyazaki M., Zaitsu M., Inada S., Ichimaru T., Hamasaki Y. (2004). Upregulation of interieukin-4 receptor by interferon-γ—Enhanced interleukin-4-induced eotaxin-3 production in airway epithelium. Am. J. Respir. Cell Mol. Biol..

[16] Mitchell C., Provost K., Niu N., Homer R., Cohn L. (2011). IFN-γ acts on the airway epithelium to inhibit local and systemic pathology in allergic airway disease. J. Immunol..

[17] Serra A., Macia A., Romero M.-P., Salvado M.-J., Bustos M., Fernandez-Larrea J., Motilva M.-J. (2009). Determination of procyanidins and their metabolites in plasma samples by improved liquid chromatography-tandem mass spectrometry. J. Chromatogr. B.

[18] Coleman S.L., Hurst R.D., Sawyer G.M., Kruger M.C. (2016). The in vitro evaluation of isolated procyanidins as modulators of cytokine-induced eotaxin production in human alveolar epithelial cells. J. Berry Res..

[19] Pierini R., Kroon P.A., Guyot S., Ivory K., Johnson I.T., Belshaw N.J. (2008). Procyanidin effects on oesophageal adenocarcinoma cells strongly depend on flavan-3-ol degree of polymerization. Mol. Nutr. Food Res..

[20] Mackenzie G.G., Delfino J.M., Keen C.L., Fraga C.G., Oteiza P.I. (2009). Dimeric procyanidins are inhibitors of NF-κB-DNA binding. Biochem. Pharmacol..

[21] Garcia G., Godot V., Humbert M. (2005). New chemokine targets for asthma therapy. Curr. Allergy Asthma Rep..

[22] Zhou L., Kawate T., Liu X., Kim Y.B., Zhao Y., Feng G., Banerji J., Nash H., Whitehurst C., Jindal S. (2012). STAT6 phosphorylation inhibitors block eotaxin-3 secretion in bronchial epithelial cells. Bioorg. Med. Chem..

[23] Hebenstreit D., Luft P., Schmiedlechner A., Duschl A., Horejs-Hoeck J. (2005). SOCS-1 and SOCS-3 inhibit IL-4 and IL-13 induced activation of Eotaxin-3/CCL26 gene expression in HEK293 cells. Mol. Immunol..

[24] Lim E., Rothenberg M.E. (2014). Demethylation of the human eotaxin-3 gene promoter leads to the elevated expression of eotaxin-3. J. Immunol..

[25] Verstraeten S.V., Keen C.L., Schmitz H.H., Fraga C.G., Oteiza P.I. (2003). Flavan-3-ols and procyanidins protect liposomes against lipid oxidation and disruption of the bilayer structure. Free Radic. Biol. Med..

[26] Rice-Evans C.A., Miller N.J., Paganga G. (1996). Structure-antioxidant activity relationships of flavonoids and phenolic acids. Free Radic. Biol. Med..

[27] Pietta P.G. (2000). Flavonoids as antioxidants. J. Nat. Prod..

[28] Feldman M., Tanabe S., Howell A., Grenier D. (2012). Cranberry proanthocyanidins inhibit the adherence properties of *Candida albicans* and cytokine secretion by oral epithelial cells. BMC Complement. Altern. Med..

[29] Gupta K., Chou M.Y., Howell A., Wobbe C., Grady R., Stapleton A.E. (2007). Cranberry products inhibit adherence of P-fimbriated Escherichia coli to primary cultured bladder and vaginal epithelial cells. J. Urol..

[30] Howell A.B., Reed J.D., Krueger C.G., Winterbottom R., Cunningham D.G., Leahy M. (2005). A-type cranberry proanthocyanidins and uropathogenic bacterial anti-adhesion activity. Phytochemistry.

[31] Carvalho E., Povoas M.J., Mateus N., de Freitas V. (2006). Application of flow nephelometry to the analysis of the influence of carbohydrates on protein-tannin interactions. J. Sci. Food Agric..

[32] Verstraeten S.V., Fraga C.G., Oteiza P.I. (2015). Interactions of flavan-3-ols and procyanidins with membranes: Mechanisms and the physiological relevance. Food Funct..

[33] Sirk T.W., Brown E.F., Friedman M., Sum A.K. (2009). Molecular binding of catechins to biomembranes: Relationship to biological activity. J. Agric. Food Chem..

[34] Helmreich E.J.M. (2003). Environmental influences on signal transduction through membranes: A retrospective mini-review. Biophys. Chem..

[35] Tsuchiya H. (2015). Membrane interactions of phytochemicals as their molecular mechanism applicable to the discovery of drug leads from plants. Molecules.

[36] Verstraeten S.V., Hammerstone J.F., Keen C.L., Fraga C.G., Oteiza P.I. (2005). Antioxidant and membrane effects of procyanidin dimers and trimers isolated from peanut and cocoa. J. Agric. Food Chem..

[37] Zhu W., Xiong L., Peng J.M., Deng X.Y., Gao J., Li C.M. (2016). Structure-dependent membrane-perturbing potency of four proanthocyanidin dimers on 3T3-L1 preadipocytes. J. Agric. Food Chem..

[38] Zhu W., Zou B., Nie R.Z., Zhang Y., Li C.M. (2015). A-type ECG and EGCG dimers disturb the structure of 3T3-L1 cell membrane and strongly inhibit its differentiation by targeting peroxisome proliferator-activated receptor γ with miR-27 involved mechanism. J. Nur. Biochem..

[39] Hurst S.M., McGhie T.K., Cooney J.M., Jensen D.J., Gould E.M., Lyall K.A., Hurst R.D. (2010). Blackcurrant proanthocyanidins augment IFN-γ-induced suppression of IL-4 stimulated CCL26 secretion in alveolar epithelial cells. Mol. Nutr. Food Res..

[40] Nyanhanda T., Gould E.M., McGhie T., Shaw O.M., Harper J.L., Hurst R.D. (2014). Blackcurrant cultivar polyphenolic extracts suppress CCL26 secretion from alveolar epithelial cells. Food Funct..

