# Supplementary material for: Procyanidin A2 Modulates IL-4-Induced CCL26 Production in Human Alveolar Epithelial Cells
Source: Int J Mol Sci. 2016 Nov 12;17(11):1888. doi: 10.3390/ijms17111888 (PMC5133887; doi:10.3390/ijms17111888)
Supplement: Supplementary file 1 [file ijms-17-01888-s001.pdf]

# Supplementary Material: Procyanidin A2 Modulates IL-4-Induced CCL26 Production in Human Alveolar Epithelial Cells

**Sara L. Coleman, Marlena C. Kruger, Gregory M. Sawyer and Roger D. Hurst**

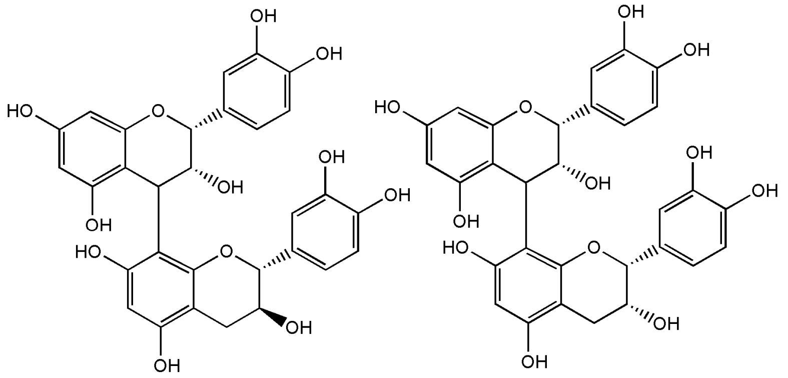


procyanidin B1
epicatechin-(4β-8)-catechin

procyanidin B2
epicatechin-(4β-8)-epicatechin

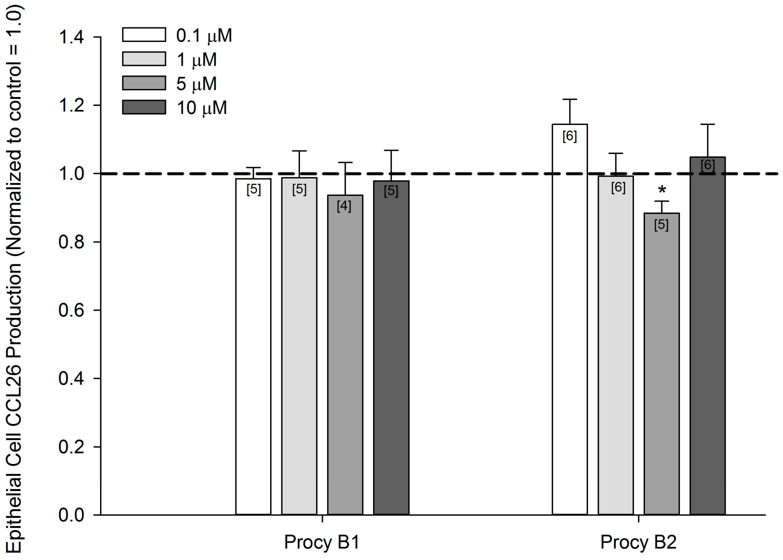


**Figure S1.** Procyanidin B1 and B2 effects on IL-4-stimulated CCL26 production. A549 cells were seeded at $5 \times 10^5$ in 12-well plates, serum starved for 24 h and incubated with control DMSO or a range of procyanidin B1 (procy B1) and procyanidin B2 (procy B2) concentrations for 6 h, washed and then stimulated with 5 ng/mL IL-4 for 24 h. Collected supernatants were measured for CCL26 by ELISA. Results are expressed as mean ± SEM normalized to control = 1.0, *n* = 4–6 separate experiments. Brackets, [*n*] indicate number of experiments for each data point. * $p < 0.05$. 5 μM procyanidin B2 reduced CCL26 production 12% compared to control.
